# Receptor Discordance in Metastatic Breast Cancer; a review of clinical and genetic subtype alterations from primary to metastatic disease

**DOI:** 10.1007/s10549-024-07431-6

**Published:** 2024-08-01

**Authors:** Gavin P. Dowling, Stephen Keelan, Nicola S. Cosgrove, Gordon R. Daly, Katie Giblin, Sinead Toomey, Bryan T. Hennessy, Arnold D. K. Hill

**Affiliations:** 1 Medical Oncology Lab, Department of Molecular Medicine, Royal College of Surgeons in Ireland, Dublin, Ireland; 2 The Department of Surgery, Royal College of Surgeons in Ireland, Dublin, Ireland; 3 The Department of Surgery, Beaumont Hospital, Dublin, Ireland

**Keywords:** Breast cancer, Metastases, Receptor discordance, Subtype switching, Intrinsic molecular subtype

## Abstract

**Purpose:**

Receptor and subtype discordance between primary breast tumours and metastases is a frequently reported phenomenon. The aim of this article is to review the current evidence on receptor discordance in metastatic breast cancer and to explore the benefit of performing a repeat biopsy in this context.

**Methods:**

Searches were undertaken on PubMed and Clinicaltrials.gov for relevant publications and trials.

**Conclusion:**

The current guidelines recommend offering to perform a biopsy of a metastatic lesion to evaluate receptor status. The choice of systemic therapy in metastatic disease is often based on the receptor status of the primary lesion. As therapeutic decision making is guided by subtype, biopsy of the metastatic lesion to determine receptor status may alter treatment. This article discusses discordance rates, the mechanisms of receptor discordance, the effect of discordance on treatment and survival outcomes, as well as highlighting some ongoing clinical trials in patients with metastatic breast cancer.

## Introduction

Breast cancer is the most common cancer and the second leading cause of cancer-related mortality among women worldwide. Despite advances in treatment, approximately 30% of patients diagnosed with early-stage breast cancer develop metastases during their lifetime [1]. Breast cancer is subtyped into different molecular groups based on immunohistochemistry (IHC) testing for the expression of the two hormone receptors: estrogen receptor (ER) and progesterone receptor (PR) along with human epidermal growth factor receptor 2 (HER2) from formalin fixed paraffin embedded (FFPE) tissue samples taken from core biopsy of the primary breast tumour [2, 3]. The ER, PR and HER2 status of the tumour are used to individualise treatment strategies in breast cancer [4]. The choice of systemic therapy in metastatic disease is often based on the receptor status of the primary lesion. However, discordances between the receptor status of the primary tumour and metastases occur [5–9]. As therapeutic decision making is guided by subtype, biopsy of the metastatic lesion to determine receptor status may alter treatment [10, 11]. Thus, current guidelines recommend offering biopsy of a metastatic lesion to evaluate receptor status [4, 12–14]. This review will discuss the rates of receptor discordance and its clinical significance.

## Discordance rates

Receptor discordance in metastatic breast cancer describes the phenomena where the ER, PR or HER2 receptor status of the primary tumour subtype has changed when re-testing is performed by immunohistochemistry on the biopsy of corresponding metastatic tumour tissue. Receptor discordance between primary breast cancer and metastases has been investigated in two independent meta-analyses [6, 15]. Aurilio et al. evaluated the discordance rates for ER, PR and HER2 receptors in a total of 9926 tumours across 48 studies. The pooled discordance proportions for ER, PR and HER2 receptors were 20%, 33% and 8%, respectively [6]. A similar meta-analysis performed by Schrijver et al. had comparable results, with discordance rates analysed in 6118 tumours across 39 studies. They reported the pooled discordances rates for ER, PR and HER2 receptors as 19.3%, 30.9% and 10.3%, respectively. This study also reported the direction of conversion, as well as location-specific differences between metastases. The positive-to-negative conversion rates for ER, PR and HER2 were 22.5%, 49.4% and 21.3%, respectively. The negative-to-positive conversion rates for ER, PR and HER2 were 21.5%, 15.9% and 9.5%, respectively. In addition, ER discordance rates were higher in bone (29.3%) and brain (20.8%) than in liver (14.3%) metastases. PR discordance rates were higher in liver (47%) and bone (42.7%) than in brain (23.3%) metastases. No statistically significant differences were observed in HER2 discordance rates between metastatic sites [15].

## Intrinsic molecular subtype switching

In addition to receptor discordance by ER, PR and HER2 IHC status, several next generation sequencing (NGS) studies have also reported frequent intrinsic molecular subtype switching in breast cancer [16–20]. Intrinsic molecular subtyping is typically assigned using the PAM50 gene expression based classifier on RNA sequencing data from breast tumours [21, 22]. The intrinsic molecular subtypes are Luminal *A*, Luminal *B*, HER2-enriched (HER2-E), basal-like and normal-like. Some studies have reported the clinical subtype by IHC does not completely overlap with the intrinsic molecular subtype by NGS indicating that subtype switching may be more frequent than if only reporting on receptor discordance by IHC [16, 20, 23].

Cejalvo et al. performed targeted gene expression profiling of 123 paired primary and metastatic tumours enriched for skin, lymph node, liver and bone metastases [17]. The rate of intrinsic subtype switching identified was 55.3% in Luminal *A*, 30% in Luminal *B*, 23.1% in HER2-E and 0% in basal-like tumours. Similar to ER and PR IHC status discordance rates, subtype switching was more frequent in Luminal type tumours with 40.2% of Luminal *A* switched to Luminal *B* while 14.3% of Luminal *A/B* switched to HER2-E tumours. In a larger more recent NGS study of paired primary tumours and early-course (de novo) metastases enriched for liver, lymph node, skin and soft tissue metastases, Afitmos et al. reported intrinsic subtyping switching occurred in 36% of cases with most Luminal *A* primary breast tumours changing subtype with metastatic disease [16]. Comparable Luminal *A* to Luminal* B* or HER2-E subtype switching rates have been reported in gene expression studies of breast cancer site specific metastases including bone or brain metastases. Priedigkeit et al. had reported in ~ 36% (4/11) cases of ER+ breast cancer Luminal *A* to Luminal *B* intrinsic subtype switching in bone metastasis [24]. In breast cancer brain metastases, recurrent gene expression losses in ESR1 (ER) and gene expression gains in ERBB2 (HER2) have been attributed to Luminal (ER+) intrinsic subtype switching [18, 19]. Consistent with this, Cosgrove et al. reported intrinsic molecular subtype switching from primary breast to brain metastases for 27% (12/45) of cases, with 8 Luminal *A* patients switching to either Luminal *B*, HER2-E or basal-like subtype; 2 patients switching from normal-like or Luminal *B* to HER2-E and 2 patients with HER2-E or Luminal *A* to basal-like subtype [20].

## Effect of discordance on treatment and survival outcomes

There is conflicting data in the literature with regards to alterations in management and the survival benefit of performing a repeat biopsy of metastases. The majority of studies examining this benefit are small retrospective analyses with variability in laboratory techniques and definitions of recurrence [11, 25–27]. To address these limitations, two independent prospective studies were carried out—the DESTINY study[28] and the BRITS study [29]. A pooled analysis of these studies evaluated the clinical impact of performing biopsy of metastatic breast cancer, specifically assessing the proportion of patients who underwent a change in management based on the biopsy results [30]. Of the 289 patients who underwent biopsy of the metastasis, 14.2% had a change in management based on the results. However, these studies failed to definitively assess the effect altering treatment had on patient outcomes, and only the DESTINY trial collected data on overall survival (OS). Receptor discordance was not significantly associated with differences in OS, if treatment was modified accordingly (median OS 27.7 months vs. 30.2 months in the concordant and discordant groups, respectively). Retrospective studies have reported worse survival outcomes in patients with receptor discordance [9, 31, 32], however this may be because of inappropriate targeted therapy in discordant cases. A recent meta-analysis investigating the prognostic significance of receptor discordance showed that a loss of either ER or PR in recurrent tumours was significantly associated with worsened OS [33]. Further large prospective studies with sufficient follow up on treatment response and survival are necessary to determine the true clinical significance of repeat biopsy for patients with metastatic breast cancer.

## Mechanisms of receptor discordance

There are many possible aetiologies for receptor discordance between the primary tumour and metastasis. It has been debated whether this discordance is due to technical diagnostic issues or reflects a true biological phenomenon, and it is likely a combination of both. Firstly, technical causes must be considered, as variability occurs in the accuracy and duplicability of IHC staining [34]. Significant variations are more often described in bone metastases, at least to some degree due to the technical issues related to decalcification, which may impact on the reliability of the IHC assessment. In addition, different sampling methods, for example fine needle aspiration or core biopsy versus surgical resection of the tumour, may contribute to discordant receptor results [11, 26, 35]. Furthermore, studies based on next generation sequencing have revealed that both intra-tumour and inter-tumour heterogeneity are of greater incidence than previously thought [36]. This supports the hypothesis that receptor discordance may be evidence of clonal genome evolution [6, 37–39]. Tumour heterogeneity may also be attributable to selective pressure of therapy inducing clonal selection with the evolution of a novel tumour cell clone [40–42], or small undetected subclones in the primary tumour that only become apparent with metastatic progression [6, 39]. In a prospective study by Hilton et al., a substantial ER discordance rate was reported between the primary tumour and metastases, but there was complete concordance among metastases arising from numerous bone sites, hinting at the existence of a dominant clone dividing from the primary tumour [43]. Biological drift is another possible explanation, as newly acquired biological characteristics in the tumour microenvironment may facilitate metastatic dissemination by enabling tumour cells to travel via the circulatory and lymphatic systems [44, 45].

Studies investigating molecular determinants of intrinsic molecular subtype switching between matched primary breast and metastatic tumour samples have been severely limited [16, 46, 47]. Given that receptor discordance and intrinsic molecular subtyping occurs most significantly in Luminal type tumours, most of these studies have focused on molecular mechanisms of ER subtype switching. ER-HER2 receptor bidirectional molecular pathway cross-talk has been largely reported in the context of endocrine or anti-HER2 therapy resistance in hormone receptor (HR) and HER2-positive breast cancer [48]. It has been proposed that a change in subtype for Luminal type tumours may be as a consequence of this ER-HER2 receptor cross-talk, where ER expression can limit PI3K pathway activity affecting HER2 pathway activation, whilst HER2 overexpression, largely due to copy number amplification, can lead to loss of ER gene expression. Epigenomic, transcriptomic or genomic analysis of Luminal type primary breast tumours which metastasize have proposed loss of ESR1 (ER) gene expression due to either ESR1 (ER) hypermethylation [18], basal-like molecular features such as TP53 and/or PIK3CA mutations [16] or increased expression of FGFR4 [46] and activation of corresponding growth factor signalling pathways may be associated with Luminal *A/B* to HER2-enriched subtype switching.

## Effect of treatment on discordance

Treatment exposure may induce receptor expression loss between the primary tumour and metastasis. This may be through a direct effect [49, 50] or as a result of clonal selection [40, 42]. For example, in line with the description of ER-HER2 receptor cross-talk above, ER and HER2 may alternate as the “dominant” pathway with either endocrine or anti-HER2 targeted therapy, where selective eradication of ER/PR positive cells by hormonal therapy could result in a population of ER/PR negative cells that could subsequently metastasise [51]. In addition to this, the impact of HER2-targeted therapy on HER2 receptor conversion has been investigated. Niikura et al. found that HER2 discordance rates differed significantly based on whether patients received chemotherapy but not based on whether the patients received trastuzumab [52]. However, other authors have not found neoadjuvant chemotherapy or trastuzumab to be associated with significant changes of HER2 status between primary and metastatic breast cancer [29, 53, 54]. The ChangeHER trial included 491 metastatic HER2-positive breast cancer cases treated with pertuzumab and/or T-DM1. This study found a HER2-positivity gain in 20.7% of cases, with some evidence of longer median OS in these patients, although at a not fully statistically significant extent [55].

## Future perspectives

### Liquid biopsy

Repeat biopsies are often technically difficult, invasive, costly, and limited by the fact that they provide information on mutations present only at a given time and site. A potential alternative for biopsy of metastatic lesions is the minimally invasive “liquid biopsy”, with genomic alterations of the tumour characterised by parallel sequencing of circulating cell-free tumour DNA (cfDNA) [56, 57] or circulating tumour cells (CTCs) [58]. These methods also attempt to tackle the heterogeneity of breast cancer as the cfDNA and CTCs stem from all malignant lesions within the body. While many studies have supported these methods [59–61], they are expensive and technically challenging. Additionally, CTCs may represent a different proportion of cells from the bulk metastatic tissue. Thus, serial blood samples provide an interesting alternative to repeat biopsies of metastatic lesions, however further research is needed to fully determine their potential.

### Ongoing trials

In an attempt to improve our understanding of the genetic aberrations in metastatic breast cancer, the AURORA trial was established [62]. In this large global study, high coverage targeted gene and RNA sequencing are performed on matched primary and metastatic samples to investigate tumour heterogeneity, clonal evolution, and transcriptional changes. Initial results of the trial found that over half of the patients had molecular changes that could be matched with existing targeted therapies, highlighting the potential benefit of molecular screening in metastatic breast cancer [63]. Ongoing trials, such as SAFIR02 (NCT02299999) [64] and Alliance A071701 (NCT03994796)[65], are investigating the benefit of genetic testing to guide treatment in metastatic breast cancer. The SAFIR02 Breast trial explores using genome analysis as a therapeutic decision tool, which aims at comparing a targeted treatment administered according to identified molecular abnormalities with maintenance chemotherapy [64]. The Alliance trial investigates the benefit of genetic testing in guiding treatment for patients with any solid tumours that have metastasised to the brain using targeted therapies [65].

## Conclusion

Receptor and subtype discordance between primary breast tumours and metastases is common. This article reviews the current evidence on the rate of clinical and intrinsic molecular subtype discordance, its clinical significance in terms of management and outcomes, and provides an insight into the future perspectives for patients with metastatic breast cancer.

## Data Availability

Not applicable.

## Abbreviations

cfDNA: Cell free tumour DNA
CTCs: Circulating tumour cells
ER: Estrogen receptor
FFPE: Formalin fixed paraffin embedded
HER2-E: Human epidermal growth factor receptor 2-enriched
HER2: Human epidermal growth factor receptor 2
HR: Hormone receptor
IHC: Immunohistochemistry
NGS: Next generation sequencing
OS: Overall survival
PR: Progesterone receptor
